# Medical Students and the Impostor Phenomenon: A Coexistence Precipitated and Perpetuated by the Educational Environment?

**DOI:** 10.1007/s40670-022-01675-x

**Published:** 2022-12-09

**Authors:** Thomas Franchi, Nigel Russell-Sewell

**Affiliations:** 1 The Medical School, The University of Sheffield, Beech Hill Road, Sheffield, S10 2RX UK; 2 School of Biosciences, The University of Sheffield, Western Bank, Sheffield, UK

**Keywords:** Impostor phenomenon, Impostor syndrome, Medical students, Clance impostor phenomenon scale, Examination rankings, Pedagogical innovation

## Abstract

People who experience the impostor phenomenon have intense thoughts of fraudulence regarding their intellect or professional activities. This perception of illegitimacy leads sufferers to believe that success in their lives is attributable to some form of error. Despite the phenomenon having been thoroughly researched in a plethora of professional and educational environments, there remains a relative lack of insight into the impostor phenomenon amongst medical students. This research aimed to better understand the relationship between medical students and the impostor phenomenon, and subsequently to investigate whether their coexistence is precipitated and perpetuated by the educational environment. A cross-sectional study of medical students was conducted using a pragmatist approach, integrating quantitative and qualitative data, via a questionnaire, focus groups and interviews. The main quantitative measure used was the validated Clance Impostor Phenomenon Scale (CIPS), where higher scores indicate more marked impostor experiences. A total of 191 questionnaire responses were received, and 19 students attended a focus group or interview. The average CIPS score for the cohort was 65.81 ± 13.72, indicating that the average student had “frequent” impostor experiences. Of note, 65.4% of students were classed as having “clinically significant” impostor experiences and females scored 9.15 points higher than males on average (*p* < 0.0001). Examination rankings were frequently cited as a major contributing factor to students’ impostor feelings, and data revealed an increase of 1.12 points per decile that a student drops down the rankings (*p* < 0.05). Students’ quotes were used extensively to underpin the quantitative data presented and offer an authentic insight into their experiences. This study provides new insights and contributes to our understanding of the impostor phenomenon amongst medical students, and eight recommendations for practice are presented, which are intended to provide medical schools with opportunities for pedagogical innovation.

**Supplementary Information:**

The online version contains supplementary material available at 10.1007/s40670-022-01675-x.

## Introduction

The term “impostor phenomenon” was first coined by Pauline Clance and Suzanne Imes in 1978 and “is used to designate an internal experience of intellectual phonies” [1]. People who experience the impostor phenomenon have intense thoughts of fraudulence regarding their intellect or professional activities, even in light of verifiable and true achievements [1, 2], and are distinctly unable to internalise successful experiences [3]. This perception of illegitimacy leads sufferers of the impostor phenomenon to sincerely believe that any success in their lives can be attributed to some form of error or indeed sheer luck [4, 5]. As such, these people experience a constant and unrelenting fear that others will uncover the “truth” and expose their lack of intelligence or competence [6, 7]. Unfortunately, this prevents high achievers from recognising their successes and inhibits any positive development in self-esteem [8], therefore also blocking people from fulfilling their innate potential [9].

At the time of inception, the impostor phenomenon was postulated to be an experience encountered predominantly by high-achieving women in professional fields [1]. However, subsequent research suggests that feeling like an impostor is much more widespread in our modern meritocratic society than might be expected, with 70% of people from all genders, ages, and backgrounds reporting that they have experienced at least one episode of impostorism in their lifetimes [10]. It has now been investigated in a plethora of settings and has been demonstrated in both males and females [11, 12], as well as in populations ranging from professionals [13] to university professors [14] to medical students [15].

It has been suggested that the phenomenon can be distinguished by six dimensions [6, 16]: the “impostor cycle”; the need to be special or the best; characteristics of superman/superwoman; fear of failure; denial of ability and discounting praise; and feeling fear and guilt about success. The instrument most widely used when identifying the impostor phenomenon is the Clance Impostor Phenomenon Scale (CIPS) [6]. This twenty-item scale assesses specific attributes associated with the phenomenon, namely through the above six dimensions, and has previously been validated for this purpose [17, 18]. Higher scores are indicative of impostor characteristics [19].

### The Impostor Phenomenon in Medicine and Medical Education

The impostor phenomenon is more widely encountered in fields where intellect is highly regarded, such as in academia [20], and people who are drawn to such areas of work are likely to have perfectionist traits and operate in an achievement-oriented manner [21]. Given the elevated personal expectations and rates of perfectionism found in medicine [22], it is not surprising that the literature indicates a particularly high prevalence of phenomenon amongst those in the medical profession [23, 24]. Impostor feelings have been reported in all levels, from students [15] to surgeons [25]. Since burnout and its devastating consequences are rife amongst doctors [26], and the impostor phenomenon is a strong contributing factor to this [27, 28], it is critical to develop our understanding of the phenomenon.

A contemporary scoping review [23] allows for a partial depiction of the impostor phenomenon in medicine to be drawn. Of the nineteen included studies, the majority were conducted in the United States of America (USA) and eight involved medical students [23]. Of note was the prevalence of impostor phenomenon amongst medical students, which ranged from 22 to 60%. When investigating gender differences, three studies found statistically significantly higher rates of impostorism in women [15, 28, 29], whilst three reported no differences [30–32]. When examining contributing factors aside from gender, the authors comment that “the hierarchy in medical education and the overall culture of medicine may perpetuate feelings of [the impostor phenomenon], as asking for help and not knowing the answer can be interpreted as signs of weakness in these environments” [23].

More recent cross-sectional cohort studies add some further colour to the painting [33–35]. The first investigated medical and dental students, concluding that female gender was the only independent predictor of intense impostor experiences in their cohort (odds ratio: 1.92, 95% confidence interval: 1.12–3.27, *p* < 0.05) [33]. The latter two conclude that females scored 9% higher on measures of impostorism than males [34]. This cohort was the first in which examination performance, using the United States Medical Licensing Examination (USMLE) Step 1, was correlated with CIPS score [34]. Whilst a moderate negative correlation was found in males, no trend was seen in females.

### Study Purpose and Research Questions

There has been a recent spike in interest surrounding research on the impostor phenomenon [36]. Despite this, there remains a relative lack of insight into the phenomenon within medicine compared to other fields. In particular, an absence of focus surrounding medical students was noted and the literature shed only dim light on the impacts of the educational environment on feelings of impostorism. Therefore, the following research questions (RQs) were formulated to systematically address the unexplored challenges identified in the literature:

- RQ1—What are medical students’ experiences of the impostor phenomenon?
- RQ2—What associated experiences are encountered in the educational environment?
- RQ3—What role do examinations play in experiences of the impostor phenomenon?
- RQ4—What pedagogical changes could help prevent or mitigate such experiences?

## Methods

In light of the purpose of this study, a pragmatic methodology was chosen [37, 38], integrating both quantitative and qualitative data to create an opportunity for a “holistic understanding” of study topics [39], and to allow for “qualitative research to inform the quantitative portion of research studies, and vice versa” [38]. Based on this philosophy, a questionnaire and follow-up focus groups and interviews were used. The rationale and purpose for the use of these mixed methods was to allow the authors to explore and present any potential explanations for the quantitative findings elicited, and the logic in combining them was to provide a more robust and student-voice-based pool of evidence.

### Setting and Participants

The study population was medical students undertaking the MBChB Medicine programme at The University of Sheffield Medical School, United Kingdom (UK), providing a potential participant pool of 1384 students. Participation was voluntary and students self-selected via a *Google Form* questionnaire (Google LLC, Mountain View, CA, USA) which, along with an information sheet and consent form, was distributed to the target population in March 2020 via the medical school’s virtual learning environment. No incentive was offered, and the form closed after 16 days.

### Data Collection

A questionnaire was constructed in a manner which considered guidance from the Association for Medical Education in Europe [40]. A literature review of previous work in the field was undertaken, to identify existing scales and previously used items. Items were then developed, encompassing a variety of question types and response modes, including dichotomous, free multiple-choice, Likert-scale and open-ended white-space questions. The questionnaire was ethically approved by the university, but formal expert validation, cognitive interviews and pilot testing were felt to be beyond the scope and purpose of this study. The questionnaire, comprising 39 questions which are detailed below, was built into the *Google Form* used to recruit participants.

The questionnaire commenced with demographic questions to allow for grouped analysis. It then followed with two dichotomous questions regarding whether participants considered themselves to be a high achiever, and whether other people would consider them as a high achiever. Students were asked to report their average decile ranking in medical school examinations, alongside whether they had ever scored in the top and/or bottom 10% of a medical school examination.

Most quantitative data were collected through the CIPS scale [6]. This twenty-item Likert scale assesses attributes associated with the phenomenon via the use of fake (assesses self-doubt and perceptions of intelligence), discount (assesses inability to acknowledge praise or success), and luck (assesses attribution of success to chance or error) subscales [41]. It has been previously both validated and psychometrically examined [17, 18, 41]. The response options are as follows: not at all true (1), rarely (2), sometimes (3), often (4), or very true (5). The text of each CIPS item is available to read here: https://www.paulineroseclance.com/impostor_phenomenon.html.

Respondents were then asked a free multiple-choice question regarding associated experienced with the impostor phenomenon, which included “perfectionism”, “fear of success”, “fear of failure”, “fear of negative evaluation”, “self-criticism”, “social anxiety”, and “none of the above”. These items were chosen as they were repeatedly encountered during the literature review. This was followed by three open-ended white-space questions. The form ended with an invitation to participate in a follow-up focus group or interview. The full questionnaire items are provided as Supplementary item 1.

Students who indicated a willingness to participate were emailed a further information sheet and consent form. Both focus groups and interviews were offered, to allow for greater triangulation of data and more robust knowledge production [42]. *Google Meet* (Google LLC, Mountain View, CA, USA) was used to host sessions, which were facilitated by the first author (TF). A semi-structured conversational approach was taken and discussions explored topics found in the questionnaire responses (the topic guide is provided as Supplementary item 2). Sessions were recorded and transcribed, with participants given a pseudonym, alongside their gender and year group (e.g. LaraF5), to ensure anonymity but allow for longitudinal tracking. Transcripts were reviewed every three sessions, to assess for saturation.

### Statistical Analysis

Analysis was conducted using *IBM SPSS Statistics for Macintosh* version 26 (IBM Corp., Armonk, NY, USA) and *R: A Language and Environment for Statistical Computing* (R Foundation for Statistical Computing, Vienna, Austria).

Before analysing CIPS data, its internal consistency was assessed using Cronbach’s alpha, where *α* ≥ 0.70 demonstrates that the items correlate with one another [43]. In this dataset was *α* = 0.90, so internal consistency was very high, indicating that the scale was suitably reliable. The responses to the twenty CIPS items by each participant were combined to generate a score between twenty and one hundred. In line with the score’s interpretation guidance [6], responses were categorised into “few” experiences (CIPS ≤ 40), “moderate” experiences (CIPS 41–60), “frequent” experiences (CIPS 61–80), and “intense” experiences (CIPS > 80) of the phenomenon. Scores were further classified to determine “clinically significant” impostor experiences, as defined by a CIPS score ≥ 62 [44]. The responses to individual CIPS items were also evaluated and correlated with total CIPS scores.

Descriptive statistics are presented as mean (*μ*) ± standard deviation (SD). The strength of association between sets of variables was calculated via Pearson’s product-moment correlation coefficient (PPMCC). The statistical significance of the *r* values produced is reported by *p* values, where *p* < 0.05 was deemed statistically significant. Ordinal least squares (OLS) regression was used to determine relationships between variables in the presence of dummy variables and is reported by *t* value. Analysis of variance (ANOVA) was used to examine associations between more than two groups and is reported by *F* statistic.

When investigating demographic datapoints, if a student preferred not to say their gender, this person’s data would be included in all calculations aside from those pertaining specifically to gender associations. When investigating experiences associated with the impostor phenomenon as reported by participants in the free multiple-choice question, students were assigned a number between 0 (indicating only “none of the above”) and 6 (indicating all options aside from “none of the above”). When investigating relationships between medical school examinations and students’ experiences of the impostor phenomenon, first-year students’ responses were included for qualitative but excluded for quantitative analysis as they had not yet undertaken summative examinations. The cohort size for any quantitative data analysis referring to examination rankings is therefore 130 students.

In line with the research philosophy, qualitative data was primarily used as explanatory evidence when presenting quantitative results. Individual quotes of significance from the transcripts were categorised by one author (TF) into themes corresponding with the RQs of the study, to assess for commonly commented-upon topics or widely held viewpoints. A theme-by-theme weaving method was used to integrate the mixed data streams through the research narrative, whereby a selection of representative quotes from the relevant theme were used to provide further insight to the quantitative results.

## Results

A total of 191 questionnaire responses were logged, representing a 13.8% response rate. The mean age of respondents was 21.10 ± 2.64 years (range 18–39), with 136 (71.2%) and 54 (28.3%) participants identifying as female and male respectively. One student preferred not to say their gender. Over a third of participants were members of Black, Asian, and Minority Ethnic (BAME) groups, with the Asian population (23%) forming the largest group after White participants (64.4%). Of the students, 19 (10%) subsequently attended either a focus group (14/19, in groups of three or four) or interview (5/19). There was representation from all year groups and 13 (68.4%) were female.

### Medical Students’ Experiences of the Impostor Phenomenon (RQ1)

**Fig. 1:**
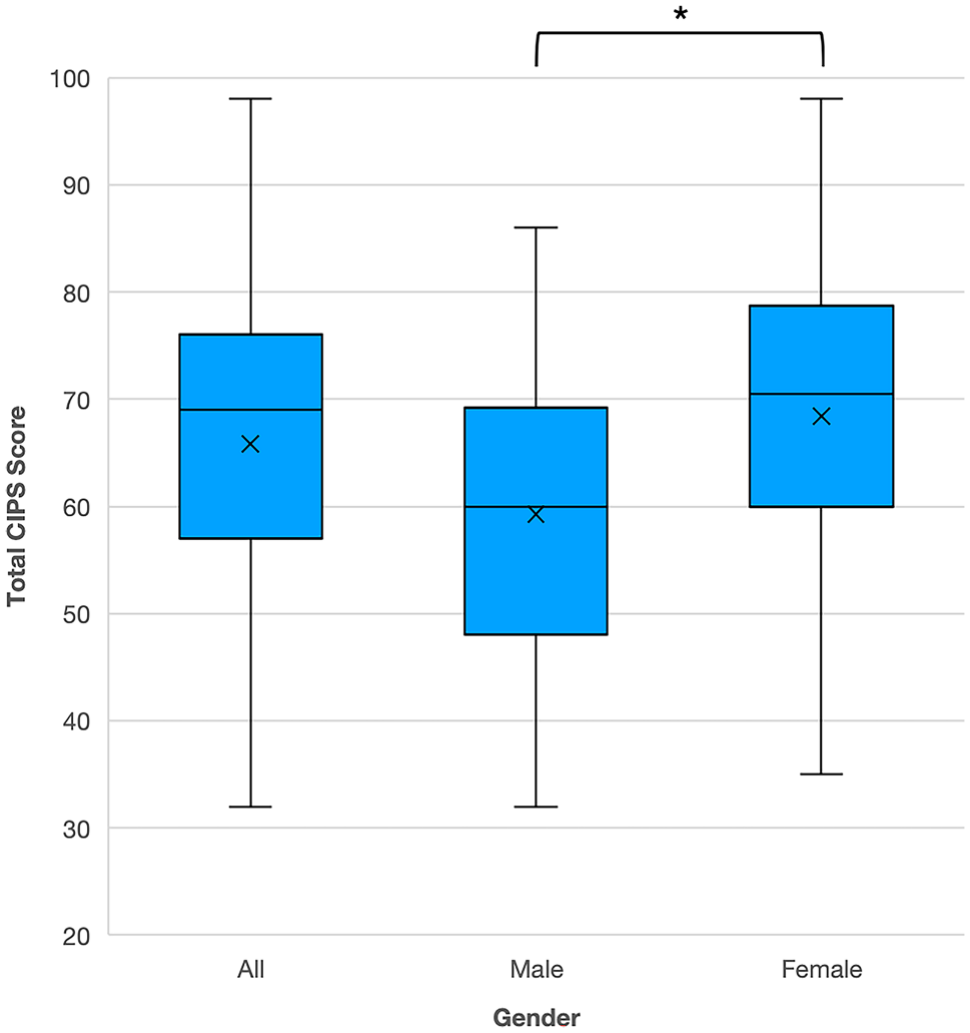
Quartile ranges of CIPS score for the whole cohort, and males and females separately. *On average, male students scored 9.15 points lower than females, *p* < 0.0001

The mean CIPS score was 65.81 ± 13.72 and a range of 32–98 was observed, so the average medical student had “frequent” impostor phenomenon. When categorised, 4.7% of respondents reported “few” experiences (CIPS ≤ 40), 29.3% reported “moderate” experiences (CIPS 41–60), 51.8% reported “frequent” experiences (CIPS 61–80), and 14.1% reported “intense” experiences (CIPS > 80). Upon comparison between the scores of males and females, the mean of the former was 59.24 ± 12.49 (range 32–86) and that of the latter was 68.56 ± 13.34 (range 35–98). This gender difference was statistically significant, with males scoring an average of 9.15 points lower on the CIPS (*t* =  − 4.34, *p* < 0.0001), Fig. 1. Observations were shared by female students which provide some insight into this difference:

“*When I compare myself to males, either young doctors or students, I definitely am a lot harsher on myself and immediately think I’m less worthy of being a doctor. I can’t brush off criticism basically, in ways I’ve noticed boys on placement can.*”—GeorgiaF5

“*If I think of the people I know who are like most chilled in their approach to like medicine and most chilled in terms of like how highly they want to rank, or in that kind of thing, generally it’s the guys that have got the more kind of chilled approach, and sort of will value work-life balance more than success.*”—MonicaF5

Further, 65.4% of the medical students were found to be in the “clinically significant” impostor category. A linear probability model found that males had a 29% lower chance of obtaining a “clinically significant” score than females (*t* =  − 3.967, *p* < 0.001). The heterogeneous effects on the CIPS score according to ethnicity were investigated, but did not elicit any differences (*F* = 0.577, *p* = 0.68).

When comparing students’ age with CIPS score, further non-significant results were found, although there was a trend of a 0.46-point reduction per year of life (*t* =  − 1.224, *p* = 0.22). However, in a direct comparison between first and final years, a first year could expect to score 9.48 points higher than a final year (*t* = 2.877, *p* < 0.01). Participants felt this indicated some form of adjustment or change in priority as students progress through medical school:

“*In first year, I was very focused on like kind of the academics, learn a lot of information and just read it all and try and absorb it. Since starting placements I’ve just been trying to focus on the clinical, you know, just trying to talk to the patients, just wanting to be good at these kinds of face-to-face interactions.*”—TaraF3

Individual responses to each of the twenty CIPS items were significantly correlated to total score, with *r* values ranging from 0.20 to 0.78 (*p* < 0.01). The highest scoring item, with an average of 4.17 ± 0.99, was Q17 (“I often compare my ability to those around me and think they may be more intelligent than I am”), whilst the lowest scoring, with an average of 2.35 ± 1.25, was Q9 (“sometimes I feel or believe that my success in my life or in my job has been the result of some kind of error”). Students’ response to Q13 (“sometimes I’m afraid others will discover how much knowledge or ability I really lack”) was the best predictor of their total score (*r* = 0.78, *p* < 0.0001), whilst their response to Q2 (“I can give the impression that I’m more competent than I really am”) was the weakest (*r* = 0.20, *p* < 0.01).

### Associated Experiences Encountered in the Educational Environment (RQ2)

In response to the multiple-choice question regarding associated experiences, “fear of failure” received the greatest response, with 83.2% of respondents aligning with it. Responses to six associated experiences were individually correlated with total CIPS score to determine their predictive significance, as detailed in Table 1. Only 11 (5.8%) respondents indicated “none of the above”, and selecting this option was a protective predictor to the total CIPS score (*r* =  − 0.30, *p* < 0.0001). Students were asked about their thoughts regarding “fear of failure”, “self-criticism” and “perfectionism”:

“*[These factors] often lead to an unnecessarily high level of stress which can negatively impact my studies in the long term. Even if others think I have done well I often think I have not and could have done better.*”—AmyF2

“*There’s always that feeling that of the high achievers, you’re not quite up there, you’re maybe one of the lower ones. The tendency to want to reach perfection means you’re quite harsh on yourself, like quite critical.*”—TaraF3

**Table 1:** Student responses to a free multiple-choice question regarding experiences associated with impostor phenomenon. (Interpretation example: In this cohort, 77.0% of students indicated that they experienced “self-criticism”, and the correlation between this experience and a student’s total CIPS score had an *r* value of 0.37 (*p* < 0.001), indicating a weakly positive relationship)

**Associated experience**	**Percentage of students indicating this associated experience**	**Correlation between experiencing this factor and total CIPS score**
Perfectionism	61.3%	0.21*
Fear of success	5.8%	0.14
Fear of failure	83.2%	0.32**
Fear of negative evaluation	40.8%	0.27**
Self-criticism	77.0%	0.37**
Social anxiety	41.9%	0.30**
None of the above	5.8%	−0.30**

^*^p < 0.01; **p < 0.001

**Fig. 2:**
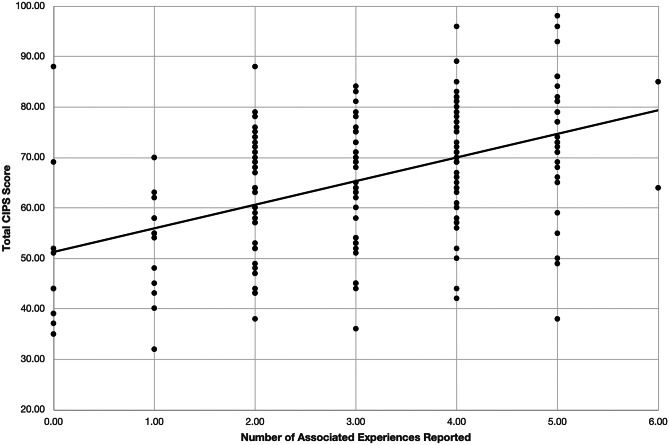
Correlation between total CIPS score and the number of associated experiences reported. Produced by PPMCC calculation (*r* = 0.49, *p* < 0.0001)

As the above experiences are related, the total number of associated factors which students identified was correlated with their total CIPS score. A statistically significant, linear relationship was found between the number of factors indicated and the total CIPS score (*r* = 0.49, *p* < 0.0001), Fig. 2.

Initial analysis of whether students consider themselves to be a high achiever (q1) and whether other people would consider them as a high achiever (q2) showed that 90.1% of students answered yes to the latter question, and 68.6% answered yes to the former. If students responded yes to q1, they could expect a 4.63-point reduction in total CIPS score (*t* =  − 2.19, *p* < 0.05), indicating this to be a protective factor. Reporting yes to q2 was also found to be a protective factor, where respondents could expect an 8.81-point reduction (*t* =  − 2.70, *p* < 0.01). Whilst not statistically significant (*p* values of 0.13–0.23), when categorised into those who undervalue themselves (“no, no”), feel undervalued (“yes, no”), are modest (“no, yes”), or are confident (“yes, yes”), feeling modest or confident was protective compared to baseline (“no, no”), with confident students receiving an 8.43-point reduction in CIPS score, Table 2.

**Table 2:** Expected total CIPS scores dependent on how students responded to questions regarding whether they consider themselves to be a high achiever (q1) and whether other people would consider them as a high achiever (q2). An OLS regression resulted in no significant differences but did reveal intriguing trends. (Interpretation example: If a student indicated yes to both q1 and q2, they might subjectively be viewed as confident, and their average CIPS score was 8.43 points lower than that of a student who indicated no to both q1 and q2, who might subjectively be viewed as undervaluing themselves.)

**Response to questions**		**This person could be seen to…**	**Average total CIPS score**	**Change in total CIPS score from baseline**
**q1**	**q2**			
No	No	Undervalue themselves	72.47	–
Yes	No	Feel undervalued	84.50	+12.03
No	Yes	Be modest	67.61	−4.87
Yes	Yes	Be confident	64.04	−8.43

### Role of Examination Rankings in Experiences of the Impostor Phenomenon (RQ3)

Whilst ranking within the top 10% of an examination was protective against total CIPS score, resulting in an average of a 2.14-point reduction, this was not significant (*t* =  − 0.825, *p* = 0.41). However, having been ranked in the bottom 10% of the cohort on at least one occasion was a statistically significant predictor of a higher total CIPS score, with students receiving a 6.08-point increase on average (*t* = 2.02, *p* < 0.05). Participants felt this perhaps indicates that examination rankings play a greater role in the impostor experiences of students who find themselves on the lower end of the scale:

“*I think rankings are the most kind of ... well like they’re really demoralising if you’re in the bottom half, but great if you’re in the top half.*”—EllenF5

**Fig. 3:**
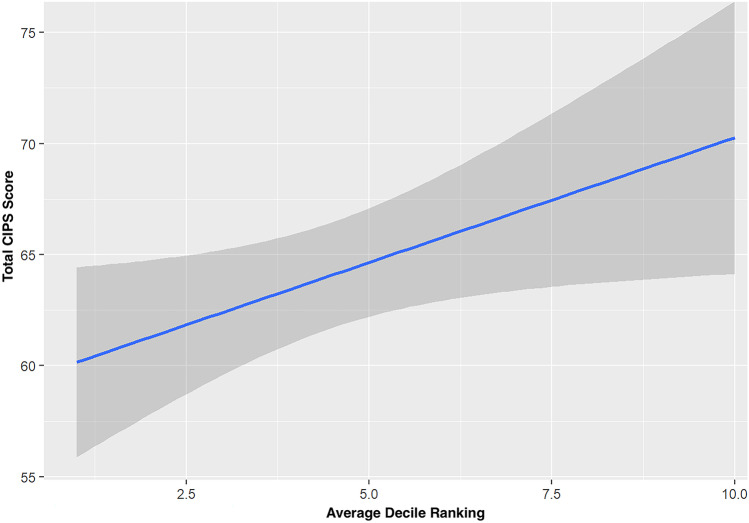
Linear regression showing the relationship between average decile ranking and total CIPS score. Dark grey shading represents 95% confidence limits

As postulated above, linear regression of students’ individual medical school decile rankings and their total CIPS score revealed a statistically significant negative relationship, indicating that the lower a students’ decile ranking, the higher their total CIPS score, Fig. 3. This result can be interpreted as a 1.12-point increase on the CIPS per decile that the student drops down the rankings (*t* = 2.17, *p* < 0.05). Conversations with participants painted a picture of this relationship, summarised here:

“*I think medical students have now been conditioned to think about their achievements purely in terms of their exam results and where they rank, and it’s no longer really about how good of a clinician they’re going to. It’s more of a ‘do you know this random fact or not?’.*”—DaveM4

The perceived importance of ranking highly was being viewed as a determining factor regarding competence as a future doctor. This translated into students questioning their clinical abilities, negatively impacting educational experiences, and perpetuating feelings of the phenomenon:

“*Having not had excellent rankings, um I very much felt [. . .] like everyone else was better than me and I’m not really good enough to be here. But you are good enough because you passed. But yeah, it’s interesting how, yeah, you start looking at things differently once you’ve looked at that ranking. Had I not looked at that ranking I would have been like, fine I’m the same as everybody else.*”—EllenF5

When asked why students perceived rankings as such an important aspect of their educational experience, many cited pressures from both the medical school and other students. Students acknowledged that rankings played some role in job allocations at the point of graduation, but many felt that their importance had been inflated to unreasonable levels:

“*There is so much pressure in first and second year to do well and get a high ranking. The medical school need to explain fully the importance, or lack of, to us in the early stages so that we realise that the world does not depend on them.*”—KellyF4

Attitudes towards teamwork, sharing knowledge and resources, and of developing collaborative bonds also seemed to be eroded by ranking students’ performance:

“*Medicine and medical school is meant to encourage teamwork and collaboration and medicine is all about kind of helping out your colleagues, yet medical school makes it so clear that you’re going be ranked against everyone else, and so you are competing against everyone else, and so nobody really wants to work together or help each other.*”—DaveM4

### Pedagogical Changes to Help Prevent or Mitigate Experiences of the Impostor Phenomenon (RQ4)

There was agreement that a more holistic system of assessment and ranking was needed. Students were keen for rankings to be based on a rounded set of attributes:

“*For learning, it would make sense if the medical school were to recognise that all the things students do on the wards […] is what students see as achievements, then they’re going to try more and are going to use it. But most students just think placement isn’t too important because that’s not what is assessed so they’ll do well in the exam and think they’re achieving.*”—ZoeF3

“*Being good with patients isn’t a tangible result, whereas with your exam results you can see exactly how you compared to everyone in the year. You can’t be like ‘oh yeah, I’m 65% good at speaking to patients’ or ‘I’m the 48th best communicator’.*”—AdamM3

Three major conclusions regarding what methods of ranking they thought would be least likely to perpetuate feelings of impostorism were reached; students wanted to be given only a decile ranking (1st to 10th) rather than an exact ranking (1st to ∼250th); told their performance percentage and its comparison to the pass mark; and be banded against a standard (70% for distinction), not each other (top 10% get distinction):

“*If we were told our exam performance by decile, then it would still let us see roughly where we rank, but would be so much less brutal than giving us an actual exact number.*”—JodyF3

“*Being able to see how well I’d passed, compared to the pass mark rather than compared to the whole year, would let me see how I was doing and let me feel good about my results regardless of my ranking. Being like ‘oh, I’m 20% better than the pass grade’ would be much nicer than being like ‘oh, I came 100th’.*”—GraceF1

“*It should be ‘this is the standard to pass, this is the standard for a merit, and this is the standard for a distinction’. If they’re going to give distinctions and stuff, then it should be based on a standard. If you’re in a bad cohort, you could get a distinction when you don’t deserve it, and the other way around.*”—PhilM4

The last student went on to describe how rankings in formative assessment might allow for healthy competition to be generated during low-stakes formative situations, rather than summative ones:

“*I think ranking for formative feedback is brilliant, so you can see how you’re doing compared to your peers . . . but in summative exams I believe that you should have to meet a level and it should be pass or fail. Um, so summative I think should only be pass or fail, but formative . . . that’s when the ranking system really comes into its own.*”—PhilM4

Finally, when asked what interventions were necessary to address the high prevalence of impostor phenomenon amongst medical students more widely, the idea of near peers sharing experiences and discussing the frank realities of the student experience at medical school appealed to participants:

“*Perhaps have people the year above come into a lecture at the start of the year and talk to the cohort, without staff in the room, about what they can expect for the coming year and become someone they can speak to that is like a close peer. People who are really keen and can share their experiences of challenges they faced, that might be beneficial.*”—DaveM4

## Discussion

This study confirmed that the impostor phenomenon amongst medical students is an undeniable endemic, and is the first to integrate quantitative and qualitative results in this manner [45]. Each of the previously outlined RQs will now be discussed in turn.

When comparing the results of RQ1 in this study with those presented by a recent scoping review [23], reasonably comparable results could be seen with regard to the average CIPS score attained and the tendency for females to score higher than men. A key divergence to note, however, was the much higher prevalence of “clinically significant” impostor phenomenon observed in this study, 65% of participants, compared to the range of 30–55% reported in previous studies [23]. This difference could be incidental, but may potentially indicate a systematic difference between medical students’ experiences of the impostor phenomenon in the UK versus the USA, where most studies reported in the review were from, via variation in factors such as educational structures, examination prevalence, or grading systems.

The data presented in RQ1 also demonstrates notable differences in CIPS scores between first- and final-year students, with the former scoring an average of 9.48 points higher. There was also a general trend of a 0.46-point reduction in CIPS score per year of life. When consulting the literature, this could be explained by the notion that impostor feelings are commonly heightened during transitional stages of life [46], such as starting university. This may also partly address why this cohort of students from the UK, who tend to start medical school aged 18 directly as school leavers [47], showed a higher prevalence of “clinically significant” impostor syndrome as compared to the studies from the USA, as medical students in the latter are postgraduates as standard with an average starting age of 24 [48], which have already had an undergraduate university experience and the adjustments that come with it. However, as mentioned above, these differences may be due to other hidden confounders. Previous authors have successfully implemented impostor-reducing interventions for first-year dental students, via the use of an “impostor video” including taped “confessionals” from former dental students and the provision of a “reminder card” containing the impostor cycle and six proposed coping mechanisms against impostorism [49]. Similar methods could be explored amongst medical students.

Further comparisons can be made when considering the analysis performed on individual CIPS items, which holds striking resemblances to a similar analysis on the responses of 112 medical students [35], where Q13 (“sometimes I’m afraid others will discover how much knowledge or ability I really lack”) was the best predictor of total CIPS score. In line with others [15, 28, 29], our study found that females were significantly more likely to experience the impostor phenomenon than their male counterparts. One female doctor described this gender imbalance as the “imposterhood” [50]. Increased societal pressures play a large role in this [1], and female-specific support mechanisms are therefore warranted.

It is clear in the literature and from the results of RQ2 that feelings of impostorism are not felt in isolation, but rather are a mixture of multiple experiences and feelings, hence why it is often referred to as a “syndrome”. This has prompted researchers to question whether the phenomenon should actually be classed as an achievement-related affective experience [51]. Previous studies investigating predictors of the phenomenon highlighted the relationships between factors such as perfectionism and social anxiety [15], and since its inception, the phenomenon has been associated with characteristics like fear of failure, fear of success, and fear of negative evaluation [6]. It is not too surprising therefore that this study reported a cumulative positive correlation between such factors and total CIPS score.

Ranking students can be seen as a clear instigator in inspiring competition between students, as seen in the results of RQ3, which can too easily translate into a distraction from learning [52]. Literature in the field also highlights the damaging implications ranking can have on students and their long-term aspirations. This draws into question the topic of comparing to a standard, not other students. Under the current system (at least at our institution), a distinction for example, which has the potential to increase a students’ likelihood of gaining the job they desire, is decided purely on whether they rank in the top 10% of their cohort. Students who deserve this achievement are therefore not being rewarded, as the system does not allow for a good cohort to be a good cohort [53].

Interestingly, the USMLE Step 1 examination will transition to a pass/fail model in 2022, moving away from the currently used framework of individual rankings. Authors have already anticipated that this is likely to benefit students, as it allows them to share knowledge without fearing comparison and will serve to strengthen teamwork, decrease stress, and undermine the impostor phenomenon [54]. Indeed, many medical schools in the USA have also already implemented a pass/fail grading method for their assessments [55], and there is increasing evidence that this provides greater satisfaction for students and less stress around exam performance [56]. Perhaps it is time for a similar approach to be adopted by UK medical schools and other institutions which still use traditional grading and ranking structures.

Recent investigation into the impact of assessments on medical students’ learning highlighted a specific need for students to understand the relevance of the assessment and its technique to their future clinical practice [57]. As part of RQ4, participants in this study indicated that this is lacking in the current framework. The above study also stressed the importance of meaningful, personalised feedback, which students in this cohort also found to be missing. A clear request from students was the implementation of channels for older students to share experiences and advice. Many viewed this as a near-peer exercise that would encourage personal and professional development alongside acting as a safety net to prevent a decline in students’ mental health [58].

### Study Limitations

Whilst a descriptive and in-depth investigation into experiences of the impostor phenomenon amongst UK medical students, this study has limitations to be considered when interpreting its results. Due to the geographical homogeneity in the sample, caution must be taken when extrapolating data from this cohort to the wider medical student context. Nevertheless, from the interpretivist perspective of this research, student quotes are completely authentic and cannot be dismissed through statistics, so do allow for translation between settings. Data was collected at a single time point as a cross-sectional study, without the possibility of prospectively monitoring the cohort. Conclusions reached about the shift in mindset as students progress through medical school could therefore be incidental. Aside from the CIPS items, the remaining questionnaire items were not validated prior to use, and so there is a potential lack of validity evidence for their use and, accordingly, the results must be interpreted with some circumspect. Further, whilst the inclusion of only 19 students in the focus groups and interviews is a potential source of self-selection bias, this number was deemed sufficient in terms of both pragmatic considerations and data saturation [59]. Indeed, concerns that only students who felt strong impostor experiences would take part in the focus groups and interviews were alleviated, as participants shared viewpoints and accounts from both “impostor” and “non-impostor” personal experiences.

Finally, the response rate of 13.8% may appear subject to potential bias, but upon comparison of demographic data to recent national statistics, the key characteristics of the wider population were captured by this sample [60]. The UK has 40,000 medical students at any point, of which females make up 55%. When considering ethnic diversity, 59.3% of students identified as White, whilst BAME students made up 36.5% of the student population. It can therefore be concluded that the study population for this research is representative and that its findings can be translated to other medical education settings. Despite this, and as with the focus groups and interview, the possibility that students who related more to impostor experiences may have been more likely to participate in the study questionnaire should also be considered. To minimise this possibility, the authors did not use the words “impostor phenomenon” in the questionnaire (Supplementary item 1), instead referring to “perceptions on their achievements”, and only raised this term directly during the focus groups and interviews.

### Implications for Research and Recommendations for Practice

To concretely measure the impact of the impostor phenomenon on medical students and to uncover factors implicated in its aetiology, studies need to be large, multicentre, prospective, and longitudinal. This research identified specific areas which warrant further investigation, including to elucidate why females appear to experience the impostor phenomenon more intensely; investigate what impact experiences of impostor phenomenon have on feelings of burnout or poor mental health; and better understand how ranking students stifles collaborative learning. There are also gaps in understanding which were not investigated, such as how a student’s education before medical school impacts their experiences at medical school; how the impostor phenomenon manifests in different personality types; and how experiences of impostorism vary depending on teaching methods and learning activities.

Finally, we offer some recommendations for practice based on the synthesis of results. They are intended to provide medical schools with suggested areas to be reviewed considering the data presented and should be considered as opportunities for pedagogical evolution and innovation. They are split into those relating to the impostor phenomenon more broadly within the medical educational environment (R1–4) and those relating specifically to the impact of examination rankings (R5–8). For each, we provide a suggestion on how this could be achieved, in italics:

- R1—Utilise this research to inform a larger investigation into the relationship between medical students and the impostor phenomenon. *This could be done as a collaboration between multiple medical schools, with the input of senior medical students.*
- R2—Acknowledge that medical students consider and perceive a wider variety of achievements than medical schools rewards and seek to uncover strategies to meaningfully encourage these. *The development of a more enriching system of praise when it comes to students’ performance both on and off the wards should be considered by medical schools’ placement support staff.*
- R3—Instigate the role of “Student Experience Mentor”, whose main responsibility would be to provide a medium for students to normalise the struggles and anxieties they face concerning the impostor phenomenon, in the form of workshop sessions with students in the year below. *These near peers could be appointed by application and work under the guidance of a lead.*
- R4—Create an anonymous online platform where students can share experiences of issues surrounding the impostor phenomenon and ask questions. *This could be monitored by the “Student Experience Mentor”, under the oversight of medical schools’ student affairs teams.*
- R5—Provide students with early and transparent guidance regarding the importance, or relative lack thereof, of medical school rankings as well as the other elements which combine to form the application scores at graduation. *This information could be distributed and discussed as career talks, in collaboration with the medical schools’ academic medicine societies.*
- R6—Launch a consultation open to all medical students regarding examination rankings, particularly to consider the use of a standard for awarding distinctions and the dissemination of deciles rather than exact rankings. *This could be done in conjunction with a trial of the proposed systems, with evaluations of both compared to each other.*
- R7—Undertake a review of the curriculum, with a focus on the constructive alignment between desired outcomes and assessment techniques used, to determine if a wider range is needed. *This could be done as a project led by senior medical students interested in medical education.*
- R8—Implement and evaluate the use of rankings in formative assessments to better inform students on their performance prior to summative assessments. *This, again, could be done as a project led by senior medical students interested in medical education.*

## Supplementary Information

Below is the link to the electronic supplementary material.

Supplementary file1 (PDF 69 kb)

Supplementary file2 (DOCX 21 kb)
