# Supplementary material for: Medical Students and the Impostor Phenomenon: A Coexistence Precipitated and Perpetuated by the Educational Environment?
Source: Med Sci Educ. 2022 Dec 9;33(1):27–38. doi: 10.1007/s40670-022-01675-x (PMC10060463; doi:10.1007/s40670-022-01675-x)
Supplement: Supplementary file 1 — Supplementary file1 (PDF 69 kb) [file 40670_2022_1675_MOESM1_ESM.pdf]

An investigation into medical students' perceptions on their achievements

You are being invited to take part in this project run by Thomas Franchi (a medical student currently intercalating the MSc Human Anatomy with Education out of phase 3a) around the experiences and feelings medical students encounter in relation to their achievements. Before you decide it is important for you to understand why this work is being done and what it will involve. Please take time to read the below Information Sheet and Consent Forms carefully and do ask if there is anything that is not clear or if you would like more information. You are also welcome to contact Dr. Nigel Russell-Sewell who is supervising this project.

Much research has been done into student perceptions on their achievements, and the consequences that this has on them. However, little has been done in relation to medical students specifically. The aim of this project is to characterise the perceptions of medical students on their achievements, with specific outcomes including the identification of potential measures to mitigate any negative impacts on the students' learning experience.

Information: https://drive.google.com/open?id=11R198BYZx-nT5HutbFteQhqFa8y18oqm
Consent: https://drive.google.com/open?id=1l9bvQYePU1XUMk9lDSxKE_hBRjqTA0M1

This project is being publicised to all medical students in all phases at The University of Sheffield Medical School, and should take between 5-10 minutes to complete. Whilst participating in this research will not benefit you directly, your responses will help to increase our understanding of the topic, and may very well be used to make recommendation for practice to The Medical School. This will have potential to impact future students' experiences. Only your Sheffield email address will be recorded, for the purpose of confirming consent. This will then be detached from your questionnaire responses so you identity will not be able to be matched to any other data. After the 30/03/2020, all email addresses will be deleted to create the final data set. No names or dates of birth will be recorded.

By ticking the below box, you are confirming that you have read and understood the above information and attached documents, give consent to the terms outlined within them, and are electronically signing the consent form.

The respondent's email (**null**) was recorded on submission of this form.
*Required

1. Email *

2. If you are happy to proceed with this survey please tick the box below: *

   Tick all that apply.
   - [ ] I have read the above information and give consent to the terms described

A bit about you

This is to help to put the data collected from the survey into context.

3. What is your age? *

4. What gender do you identify as? *

   Mark only one oval.
   - Female
   - Male
   - Prefer not to say
   - Other: ____

5. Please specify your ethnicity: *

   Mark only one oval.
   - White
   - Mixed
   - Asian
   - Black
   - Arab
   - Other: ____

6. Do you consider yourself to be a high achiever (in your life in general, not restricted to academics)? *

   Mark only one oval.
   - Yes
   - No

7. Would others consider you to be a high achiever (in your life in general, not restricted to academics)? *

   Mark only one oval.
   - Yes
   - No

Your academic background

Again, this will help to put the data collected from the survey into context.

8. Please specify your current phase: *

   Mark only one oval.
   - Phase 1
   - Phase 2a
   - Phase 2b
   - Phase 3a
   - Phase 3b
   - Phase 4
   - Intercalating

9. When you started medical school, were you an undergraduate or postgraduate? (i.e. if you intercalated/are intercalating, please still answer undergraduate) *

   Mark only one oval.
   - Undergraduate
   - Postgraduate

10. If you answered postgraduate, please specify the highest degree type which you obtained (BSc, MSc, PhD etc). If you answered undergraduate, please leave this question blank.

11. What is your average decile ranking in medical school exams to date? If you do not know exactly, please take a guess or answer with of your most recent exam decile. *

    Mark only one oval.

	1	2	3	4	5	6	7	8	9	10	
1st decile (top 10%)											10th decile (bottom 10%)

12. Have you ever received a band 5 in any medical school exams? (i.e. top 10% of students) *

    Mark only one oval.
    - Yes
    - No

13. Have you ever received a band 3 in any medical school exams? (i.e. bottom 10% of students) *

    Mark only one oval.
    - Yes
    - No

A measure of self-confidence

For each of the following, select the answer that best indicates how true the statement is for you. In order for the results to have the biggest impact, please be honest and give the first response that enters your mind rather than thinking about it at length. The statements are taken from Clance, 1985.

14. I have often succeeded on a test or task even though I was afraid that I would not do well before I undertook the task *

    Mark only one oval.
    - Not true at all
    - Rarely
    - Sometimes
    - Often
    - Very true

15. I can give the impression that I'm more competent than I really am *

    Mark only one oval.
    - Not true at all
    - Rarely
    - Sometimes
    - Often
    - Very true

16. I avoid evaluations if possible and have a dread of others evaluating me *

    Mark only one oval.
    - Not true at all
    - Rarely
    - Sometimes
    - Often
    - Very true

17. When people praise me for something I've accomplished, I'm afraid I won't be able to live up to their expectations of me in the future *

    Mark only one oval.
    - Not true at all
    - Rarely
    - Sometimes
    - Often
    - Very true

18. I sometimes think I obtained my present position or gained my present success because I happened to be in the right place at the right time or knew the right people *

    Mark only one oval.
    - Not true at all
    - Rarely
    - Sometimes
    - Often
    - Very true

19. I'm afraid people important to me may find out that I'm not as capable as they think I am *

    Mark only one oval.
    - Not true at all
    - Rarely
    - Sometimes
    - Often
    - Very true

20. I tend to remember the incidents in which I have not done my best more than those times I have done my best *

    Mark only one oval.
    - Not true at all
    - Rarely
    - Sometimes
    - Often
    - Very true

21. I rarely do a project or task as well as I'd like to do it *

    Mark only one oval.
    - Not true at all
    - Rarely
    - Sometimes
    - Often
    - Very true

22. Sometimes I feel or believe that my success in my life or in my job has been the result of some kind of error *

    Mark only one oval.
    - Not true at all
    - Rarely
    - Sometimes
    - Often
    - Very true

23. It's hard for me to accept compliments or praise about my intelligence or accomplishments *

    Mark only one oval.
    - Not true at all
    - Rarely
    - Sometimes
    - Often
    - Very true

24. At times, I feel my success has been due to some kind of luck *

    Mark only one oval.
    - Not true at all
    - Rarely
    - Sometimes
    - Often
    - Very true

25. I'm disappointed at times in my present accomplishments and think I should have accomplished much more *

    Mark only one oval.
    - Not true at all
    - Rarely
    - Sometimes
    - Often
    - Very true

26. Sometimes I'm afraid others will discover how much knowledge or ability I really lack *

    Mark only one oval.
    - Not true at all
    - Rarely
    - Sometimes
    - Often
    - Very true

27. I'm often afraid that I may fail at a new assignment or undertaking even though I generally do well at what I attempt. *

    Mark only one oval.
    - Not true at all
    - Rarely
    - Sometimes
    - Often
    - Very true

28. When I've succeeded at something and received recognition for my accomplishments, I have doubts that I can keep repeating that success *

    Mark only one oval.
    - Not true at all
    - Rarely
    - Sometimes
    - Often
    - Very true

29. If I receive a great deal of praise and recognition for something I've accomplished, I tend to discount the importance of what I've done *

    Mark only one oval.
    - Not true at all
    - Rarely
    - Sometimes
    - Often
    - Very true

30. I often compare my ability to those around me and think they may be more intelligent than I am *

    Mark only one oval.
    - Not true at all
    - Rarely
    - Sometimes
    - Often
    - Very true

31. I often worry about not succeeding with a project or examination, even though others around me have considerable confidence that I will do well *

    Mark only one oval.
    - Not true at all
    - Rarely
    - Sometimes
    - Often
    - Very true

32. If I'm going to receive a promotion or gain recognition of some kind, I hesitate to tell others until it is an accomplished fact *

    Mark only one oval.
    - Not true at all
    - Rarely
    - Sometimes
    - Often
    - Very true

33. I feel bad and discouraged if I'm not "the best" or at least "very special" in situations that involve achievement *

    Mark only one oval.
    - Not true at all
    - Rarely
    - Sometimes
    - Often
    - Very true

Other experiences you might align with

Below is a list of related feelings which the literature shows medical students sometimes experience. Please tick as many boxes as apply to you and your journey at medical school so far. For those ticked, please let us know how these have affected you, and what could be done to help prevent, minimise or mitigate this in the space provided.

34. Do you personally align with any of the following experiences (please tick as many as apply)? *

    Tick all that apply.
    - [ ] Perfectionism
    - [ ] Fear of success
    - [ ] Fear of failure
    - [ ] Fear of negative evaluation
    - [ ] Self-criticism
    - [ ] Social anxiety
    - [ ] None of the above

35. If you align with any of the above experiences, please use the below space to comment on how these things have affected you or your studies:

36. If you align with any of the above experiences, please use the below space to comment on what The Medical School could do to help prevent, minimise or mitigate this (there is potential for this research to provide recommendations to The Medical School, so your comments could have real impact):

To end

Just two final questions!

37. If you have any comments, thoughts or questions relating to the themes in this survey, please feel free to write them here:

38. Following this survey, I aim to arrange some focus groups and interviews to further explore the themes covered. As such, I am looking for volunteers to attend these. Please tick the boxes which apply to you: *

    Tick all that apply.
    - [ ] I would be happy to be contacted regarding the focus groups
    - [ ] I would be happy to be contacted regarding the one-to-one interviews
    - [ ] I do not wish to partake in a focus group or interview

39. If you ticked that you are happy to be contacted, please kindly re-provide me with your Sheffield email address (this will be separated from your responses, so I will not be able to match them):

This content is neither created nor endorsed by Google.

Google Forms
