# Supplementary material for: Medical Students and the Impostor Phenomenon: A Coexistence Precipitated and Perpetuated by the Educational Environment?
Source: Med Sci Educ. 2022 Dec 9;33(1):27–38. doi: 10.1007/s40670-022-01675-x (PMC10060463; doi:10.1007/s40670-022-01675-x)
Supplement: Supplementary file 2 — Supplementary file2 (DOCX 21 kb) [file 40670_2022_1675_MOESM2_ESM.docx]

**Focus Groups/Interviews Topic Guide:**

*This is a rough topic guide to aid in the semi-structured focus groups and interviews, which explore key topics found in the questionnaire responses.*

1. Introduction

- Introduce myself and remind participants of who I am and the reasons for undertaking this research
- Thank participants for their contributions on the questionnaire and for offering their time to take part in the focus groups.
- Explain format of the session, highlighting that contributions are optional and people are free to say as little or as much as they wish.
- Make participants aware that they cannot share identities or comments shared by participants outside of the focus group.
- Remind participants that the session is being audio recorded for transcribing.
- Obtain verbal consent to continue.

2. Initial thoughts

- Casting your mind back to the questionnaire, and feel free to bring up your response record on your email if you wish, was there anything that stood out to you or any personal reflections that you wish to share?
- What themes do you think were predominant in your approach to answering the questions?
- Did you feel any specific emotion when answering the questionnaire or did it cause you to feel a certain way?

3. Impostor phenomenon

- Who here had ever heard of impostor phenomenon/syndrome?
- Who here feels like they align to it?
- Can anyone offer a definition?
- What do you understand about its impacts or effects on students in medical school?
- Although I didn’t use the words, the questionnaire was very much cantered around impostor phenomenon.
- Does this change the way you thought about any of the questionnaire sections?
- Would anyone be happy to share any of their experiences of this in any way, or the impact it’s had on you whilst at medical school?

4. Your perceptions compared to others

- Whilst 90% of students said other people would consider them as high achievers, only 69% of students considered themselves high achievers, why do you think this is?
- When I calculated your scores of impostor phenomenon based on responses, 14% of students were classed as having intense experiences that regularly interfere in their life. Does this surprise you?
- “I often compare my ability to those around me and think they may be more intelligent than I am”, 49% of students thought this statement was very true. Do you have any thoughts on why medical students doubt themselves so much?

5. Rankings

- Moving on from above, a common theme in the open discussion areas of the questionnaire related to the medical school ranking us after exams.
- What impacts do you think rankings have on your perceptions of your achievements?
- What about your mental health?
- Share a personal experience of my high OSCE percentage but average rank.
- Do rankings bring about a sense of impostor that you weren’t aware of before?
- Students often spoke about fear of failure, self-criticism, and perfectionism. Would you have these same issues and concerns if there were no rankings?
- Would there be the same amount of pressure and competition if no rankings?
- Do the graphs which the medical school give us to show our position in the cohort make things better or worse?

6. Culture of medicine

- Impostor phenomenon is classically seen in higher achievers and medical school is full of those. Do you think the medical school culture and that of medicine more widely needs to change to account for this?
- “Perfection is not something achievable in medicine and this is a fact that is hard to deal, because I feel like I have been striving for perfectionism my whole life.” What are your thoughts on this statement?

7. Different stages in medical school

- Do you think your doubts or perceptions on achievement change throughout medical school? For example, learning theory versus speaking to patients.
- Do the upper years have any reflections or advice to the lower years?
- Do the lower years have any concerns which upper years don’t experience?

8. What can medical school do

- Is it as simple as remove rankings?
- What else needs to be done?
- Some students spoke about not knowing what’s important

9. Covid-19

- Many of you might have now automatically passed your year or will only have to sit formative exams. Is it possible this might impact on students’ feelings of being an impostor?
- What impact might this have on rankings?
- Do you feel that the missed teaching/placement is going to negatively impact your self-belief that you can be a doctor?

10. Close

- Thank everyone for their valuable time and responses.
- Does anyone have any other comments, thoughts, reflections, or questions they’d like to raise?
- Inform participants that I will stay on the call until everyone has left, so if anyone wants to stay after to discuss something individually, that is fine.
